# Characterization of PLA/PCL/Green Mussel Shells Hydroxyapatite (HA) Biocomposites Prepared by Chemical Blending Methods

**DOI:** 10.3390/ma15238641

**Published:** 2022-12-04

**Authors:** Rifky Ismail, Tezara Cionita, Yin Ling Lai, Deni Fajar Fitriyana, Januar Parlaungan Siregar, Athanasius Priharyoto Bayuseno, Fariz Wisda Nugraha, Rilo Chandra Muhamadin, Agustinus Purna Irawan, Agung Efriyo Hadi

**Affiliations:** 1 Department of Mechanical Engineering, Faculty of Engineering, Diponegoro University, Semarang 50275, Indonesia; 2 Center for Biomechanics, Biomaterial, Biomechatronics, and Biosignal Processing (CBIOM3S), Diponegoro University, Semarang 50275, Indonesia; 3 Department of Mechanical Engineering, Faculty of Engineering and Quantity Surveying, INTI International University, Nilai 71800, Malaysia; 4 Department of Mechanical Engineering, Faculty of Engineering, Universitas Negeri Semarang, Semarang 50229, Indonesia; 5 College of Engineering, Universiti Malaysia Pahang, Gambang 26300, Malaysia; 6 Department of Mechanical Engineering, Faculty of Engineering, Universitas Tarumanagara, Jakarta Barat 11440, Indonesia; 7 Mechanical Engineering Department, Faculty of Engineering, Universitas Malahayati, Bandar Lampung 35153, Indonesia

**Keywords:** biocomposites, hydroxyapatite, PLA, PCL, bioceramic, biopolymer

## Abstract

Recently, there has been an increase in the number of studies conducted on the process of developing hydroxyapatite (HA) to use in biocomposites. HA can be derived from natural sources such as bovine bone. The HA usage obtained from green mussel shells in biocomposites in this study will be explored. The research goal is to investigate the composition effect of biomaterials derived from polycaprolactone (PCL), polylactic acid (PLA), as well as HA obtained from green mussel shells with a chemical blending method on mechanical properties and degradation rate. First, 80 mL of chloroform solution was utilized to immerse 16 g of the PLA/PCL mixture with the ratios of 85:15 and 60:40 for 30 min. A magnetic stirrer was used to mix the solution for an additional 30 min at a temperature and speed of 50 °C and 300 rpm. Next, the hydroxyapatite (HA) was added in percentages of 5%, 10%, and 15%, as well as 20% of the PLA/PCL mixture’s total weight. It was then stirred for 1 h at 100 rpm at 65 °C to produce a homogeneous mixture of HA and polymer. The biocomposite mixture was then added into a glass mold as per ASTM D790. Following this, biocomposite specimens were tested for their density, biodegradability, and three points of bending in determining the effect of HA and polymer composition on the degradation rate and mechanical properties. According to the findings of this study, increasing the HA and PLA composition yields a rise in the mechanical properties of the biocomposites. However, the biocomposite degradation rate is increasing.

## 1. Introduction

Traffic accidents are a major problem and they increase every year, especially in Indonesia [1]. As per the Ministry of Health, approximately 8 million people in Indonesia encounter bone fractures, of which 46.2% are derived from traffic accidents [2].

Note that bone fractures require good bone surgery management. One of the techniques for handling bone surgery is internal fixation. This procedure is performed by pressing the fixation device against the bone [3], with metals and alloys dominating the market for biomaterials for bone repair. This is because the material’s price is relatively low and possesses excellent mechanical properties. However, the use of metal implants requires a second surgical procedure for implant removal, which can increase the cost of treatment [4]. Other than this, a metallic surface’s ability to release ions, interact chemically with bodily acids and enzymes, and have a high elastic modulus can all result in toxicity, stress shielding, and oxidative or galvanic corrosion, which can lead to bone resorption and implant failure [5,6,7,8]. This has prompted researchers to find substitutes for metal implants by using other materials such as polymers, ceramics, and composites.

The use of polycaprolactone (PCL) and polylactic acid (PLA) as biopolymers has been widely seen in medical applications. This is because both polymers have been recognized by the United States Food and Drug Administration (USFDA) as the most researched polyesters, given their ease of processing and their tunability in regards to mechanical strength properties, thermal transition, and crystallinity [9]. The lactic acid oligomers and monomers that are produced during the degradation of PLA are entirely absorbed by living things. Apart from this, PLA also possesses high processability and mechanical resistance. PLA’s crystalline state can be entirely amorphous (non-crystalline) or up to 40% crystalline. The melting temperature (T_m_) of PLA ranges between 130 and 180 °C, while its glass transition temperature (T_g_) ranges between 50 and 80 °C [10]. PCL has good solubility with other polymers, minimal viscosity, and hydrophobic characteristics, in which the molecular weight and level of crystallinity affect the physical and mechanical characteristics. The glass transition temperature (T_g_) of PCL is approximately −60 °C, whereas the T_m_ ranges from 50 to 60 °C. Note that PCL has a high degree of crystallinity (between 30 and 60%) [10].

The adsorption and degradation kinetics of PCL are substantially stagnant compared to other aliphatic polyesters, which may restrict its use in some applications. In addition, the weak mechanical properties of PCL restrict its application as a scaffold to substitute hard tissue, relying on the preparation procedure and molecular weight [11]. The poor cell adhesion and slow degradation of PLA, on the other hand, may be attributed to its in vivo inflammation, biological inertness, and hydrophobicity, which is produced by the acidic degradation product [12]. Furthermore, PLA is a brittle material that degrades quickly in body fluids. Therefore, the addition of PCL to PLA is used to minimize brittleness and extend the degradation time of the PLA. Subsequently, PLA and PCL are blended to make copolymers that have good qualities for tissue engineering, for instance, being biocompatible, biodegradable, and non-toxic [10,13,14].

The main inorganic component of human hard tissue mostly consists of hydroxyapatite (HA), which has the chemical formula Ca_10_(PO_4_)_6_(OH)_2_. In the biomedical industry, HA can be utilized for drug delivery systems, implant coatings, soft tissue repair, bone fillers, as well as scaffolds for bone tissue engineering [15,16,17]. Nevertheless, the limited mechanical strength of HA prevents it from being used widely, especially as an implant material. Pure HA has flexural strengths, both tensile and compressive, ranging from 38 to 250 MPa, 38 to 300 MPa, as well as 120 to 150 MPa, accordingly [18].

In order to obtain the necessary qualities, various types of biomaterials are combined to create biocomposites. For example, the combination of a polymer matrix (PLA and PCL) with bioactive minerals (HA) produced a biocomposite material with better mechanical properties and prosthesis integration [19]. As a result, HA and its polymer-based composites have seen widespread use in orthopedic and dental applications. In addition, these biocomposites have important applications in drug delivery, cell and porous scaffolds for tissue engineering, bone screws, prostheses, bone graft, cardiovascular implants, cell culture substrates, spinal cages, anchors, vascular grafts, sutures, skin and tendon healing, antimicrobial agents, cancer therapy, and medical tools [11,12,20,21,22,23,24,25,26].

An extensive amount of research on biocomposites has been carried out. For example, Hassanajili et al. [27] utilized PLA/PCL/HA as the biocomposite material. The biocomposite with a PLA/PCL 70/30 and a 35% HA composition had the greatest results in this investigation. The composition has a Young’s modulus of 1.35 MPa, a porosity of 77%, as well as an average pore size of 160 μm. MG63 cells were also used to examine the generated biocomposite in vitro. Other than this, the evaluation of cytotoxicity demonstrates that cells may exist and propagate in the human body. In regard to viability, biocompatibility, and osteoinduction properties, the outcomes demonstrated that the biocomposite within this ratio had excellent properties. Further research performed by Pitjamit et al. [14] utilized PLA/PCL/HA as biocomposite materials for 3D printing filament fabrication. The HA occurrence in the PLA/PCL/15HA mixture yields the greatest compressive strength (82.72 ± 1.76 MPa). Furthermore, it is observed that HA also produced higher bone cell proliferation.

A bioactive hybrid composite is demonstrated by integrating a bioactive phase, for instance, HA, into a polymer matrix. This composite can significantly increase hydrophilicity, mechanical strength, and biocompatibility. The increased differentiation and proliferation of bone cells caused by HA nanoparticles, as well as the excessive deposition of calcium containing minerals in the scaffold, speed up the production of new bone tissue [27]. However, most studies still use HA from bovine bones. The utilization of green mussel shells as waste utilization is still rarely adopted. This study investigated the synthesis of biocomposites derived from PLA, PCL, and HA from green mussel shells. Furthermore, this study was conducted to determine the effect of the composition of PLA, PCL, and HA on the physical, mechanical, and degradation properties of the biocomposite.

## 2. Materials and Methods

### 2.1. Materials

**Figure 1:**
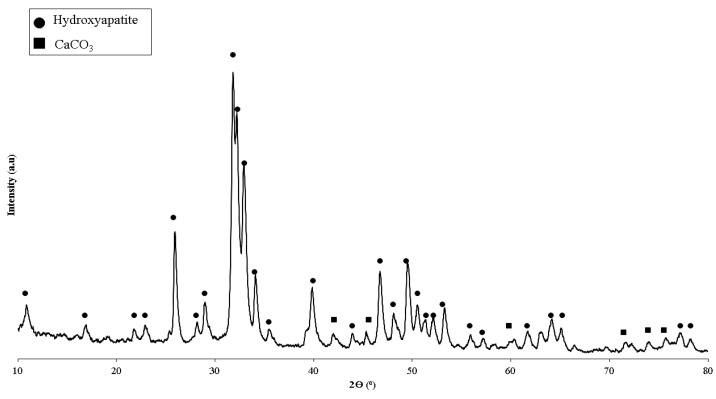
XRD diffraction on the hydroxyapatite of green mussel shells [32].

The manufacture of biocomposites utilizing the chemical blending method in this study refers to research that has been carried out previously [28]. The materials used were polylactic acid (PLA) and polycaprolactone (PCL) filaments with a diameter of 1.75 mm. The PLA and PCL filament employed was created by SUNLU Industrial Co., Ltd., (Zhuhai, China). Table 1 depicts the filament specifications. Hydroxyapatite (HA) obtained from green mussel shells was retrieved from past studies conducted by CBIOM3S, Diponegoro University, Semarang, Indonesia. The synthesis of HA was obtained from the precipitated calcium carbonate (PCC) of green mussel shells [29]. X-ray diffraction (XRD) test results on HA are shown in Figure 1. The findings of the XRD analysis revealed that the HA derived from the CBIOM3S Laboratory was the dominating phase, with a small quantity of CaCO_3_ present.

**Table 1:** PLA and PCL filament specifications.

Parameters	Materials	
	PLA [30]	PCL [31]
Wire diameter (mm)	1.75	1.75
Print temperature (°C)	180–200	70–100
Melting point (°C)	165–180	58–62
Density (g/cm^3^)	1.24	1.28
Elongation at break (%)	4	560
Melt flow index (g/10 min)	7–9	3–5
Pull force (Kgf)	11–16	2
Water absorption (%)	0.5	0.2

### 2.2. Fabrication of Specimen Test and Testing

First, 16 g of PCL and PLA (according to Table 2) were dissolved for 30 min in 80 mL of chloroform solution. The biopolymer was then mixed with a magnetic stirrer for 30 min at 300 rpm and 50 °C in a beaker sealed with aluminum foil to produce a PCL and PLA solution that was transparent and uniform. Next, the stirring procedure was continued by adding green mussel shell HA in proportions of 5%, 10%, 15%, and 20% of the biopolymer mixture’s total weight (16 g). Finally, the HA powder was added to the biopolymer mixture and stirred at 100 rpm and 65 °C for 1 h to produce a homogeneous mixture of polymer and HA.

**Figure 2:**
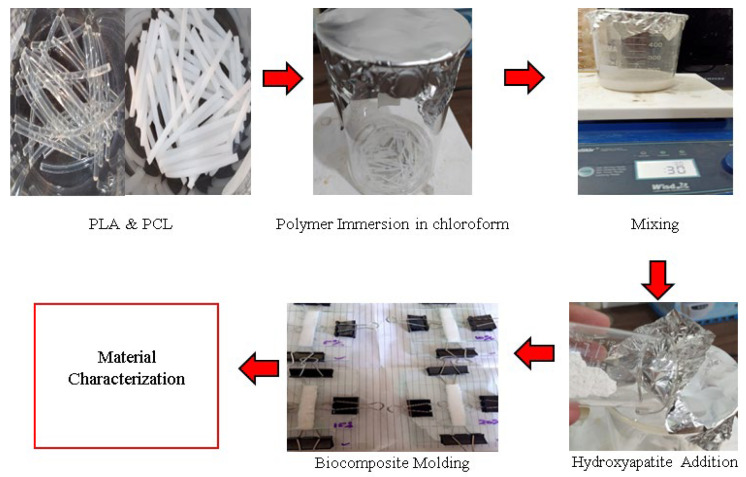
Experimental setup.

The temperature was raised to 65 °C to speed up the evaporation process of chloroform because the boiling point of chloroform is 61 °C. After 1 h of stirring with an open beaker, the chloroform evaporated, and the mixture thickened to form a paste. After that, the paste was poured into a mold made of glass. The mold followed the ASTM D790 specimen size. The drying process of the biocomposite in the mold was conducted at room temperature for two days until the chloroform completely evaporated and nothing remained on the specimen. After the chloroform evaporated, the specimen dried and hardened so that it could be removed from the mold. In order to eliminate any remaining pores in the specimen, the biocomposite was immersed in distilled water for 24 h before testing, also known as the immersion procedure. The setup for the experiment is shown in Figure 2.

The density of the final biocomposite was measured using density testing. An electronic density meter (DME 220 series) from Vibra Canada Inc. (Mississauga, ON, USA). was used to conduct density testing in accordance with ASTM 792-08. In addition, the biocomposite’s flexural modulus and flexural strength were evaluated using the three points bending test in accordance with ASTM D790. Finally, the biodegradable characteristics of the generated biocomposite were also determined using the degradable test. In accordance with previous work [28], the composite specimen was subjected to an immersion test using a 3.5%wt NaCl solution to detect its degradation behavior. A NaCl solution with a concentration of 0.85 wt.% or 0.9 wt.% has the same osmolality as human plasma and is considered a simple isotonic solution. In biomedical applications, NaCl solution has been extensively utilized for assessing the degradation and corrosion of implant materials. Unlike cell culture conditions containing protein blends, NaCl solutions have no risk of contamination and are easy enough to estimate the degradation of implant materials. The NaCl solution is useful for a variety of corrosion and degradation studies on implant materials due to its simplicity and low risk of contamination generated by the development of microbial life. The complexity of real body fluids cannot be represented by tests in NaCl solutions. However, compared to more complex media such as Hank’s balanced salt solution (HBSS) and Minimum Essential Medium (MEM), NaCl solution is the most aggressive medium that is beneficial for differentiating the intrinsic corrosion susceptibility properties of the metallic substrates [33,34,35].

By scanning the samples with infrared light, the Fourier transform infrared (FTIR) method was employed to assess the inorganic, organic, and polymeric compounds. Changes in the material composition can be seen in variations in the absorption bands’ characteristic pattern. In this study, the FTIR test used Spectrophotometer FTIR Perkin Elmer Spotlight 400 Frontier (Waltham, MA, USA) with the recorded region of 4000 to 400 cm^−1^ in order to identify biocomposite specimens. A non-destructive method for examining the structure of crystalline materials utilized an X-ray diffraction (XRD) analysis to identify the crystalline phases in a material by examining its crystal structure. The PLA/PCL/HA biocomposites samples were identified by X-ray diffraction using a Shimadzu XRD-7000 diffractometer (Kyoto, Japan) at 40 kV with a current of 30 mA and Cu Kα radiation (λ = 0.15406 nm). Diffractograms were obtained between (2θ) 10° and 90° with a step of 0.02 and a scanning velocity of 1°/min. Furthermore, the surface morphology of the PLA/PCL/HA biocomposites was inspected by utilizing a scanning electron microscope (SEM) (JSM-6510, JEOL, Tokyo, Japan) at a 15 kV accelerating voltage. A high sensitivity backscattered electron detector is installed on the bottom of the objective lens to create composition images, topography images, and shadow images.

## 3. Results and Discussion

**Figure 3:**
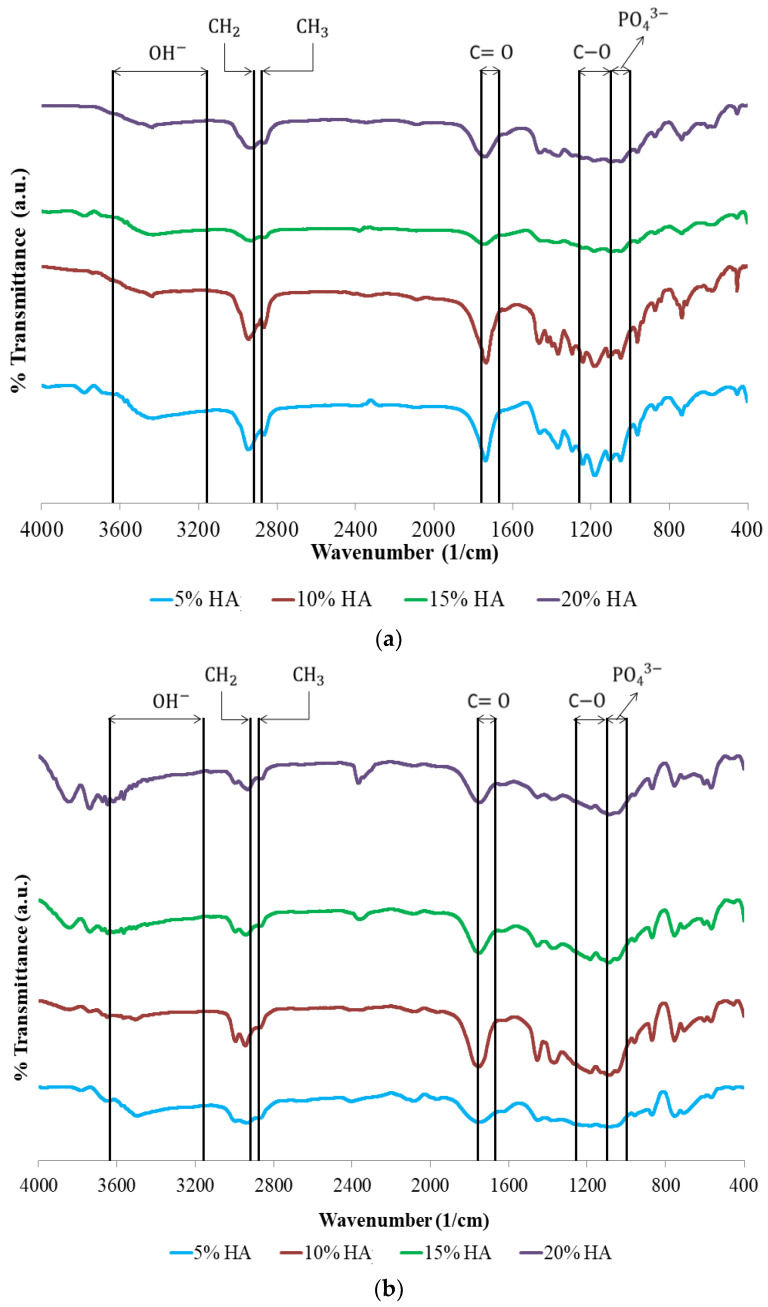
FTIR spectra of biocomposites of (**a**) PLA40/PCL60 and (**b**) PLA85/PCL15 at various concentrations of HA.

Figure 3 displays the samples’ FTIR spectra. In this study, the obtained biocomposites formed functional groups with wavenumbers that were not much different.

The presence of C−O, C=O, and CH_3_ peaks in the biocomposite specimens indicated the existence of polylactic acid (PLA). Meanwhile, C−O, C=O, and CH_2_ peaks reflect polycaprolactone (PCL) content in the biocomposite. The presence of PO_4_^3−^ and OH− peaks in the biocomposite specimens suggests the existence of hydroxyapatite (HA).

The PO_4_^3−^ stretch band around 1000–1156 cm^−1^ and OH− stretch peak at 3160–3640 cm^−1^ are depicted in the hydroxyapatite (HA) spectrum. In this study, all of the biocomposite specimens showed O–H stretch with weak intensity in the range of 3160–3640 cm^−1^. However, Figure 3 shows the presence of O–H bending with a fairly strong intensity in the range of 1440–1395 and 950–910 cm^−1^ [36,37,38].

A peak at a wavelength of 2915–2940 cm^−1^ signifies CH_2_ asymmetric stretch. In contrast, the peak at 2860–2880 cm^−1^ indicates a CH_3_ symmetrical stretch. Moreover, the C=O stretch and C−O stretch are examined at 1670–1760 cm^−1^ and 1000–1100 cm^−1^, accordingly [27,39,40]. According to the FTIR test results, PLA, PCL, and HA did not produce new peaks in the spectrum. This demonstrates that PLA, PCL, and HA mechanically bond. This happens because PLA is relatively hydrophobic, hence the PLA surface is unable to chemically bond with the HA surface [41]. The findings of this study are consistent with Shojaei et al. [39]. According to the researchers, no new peaks in the biocomposite spectrum were discovered due to the limited contact between the two polymers, which are fully incompatible [39]. The carbonyl group’s location in the biocomposite moved to a lower wavelength, according to FTIR measurements. This is due to the interaction of the carbonyl groups of PCL and PLA with the hydroxyl groups of HA in the biocomposite. These results indicate that the HA particles are dispersed between the two polymer phases [27,39,40,41,42].

**Figure 4:**
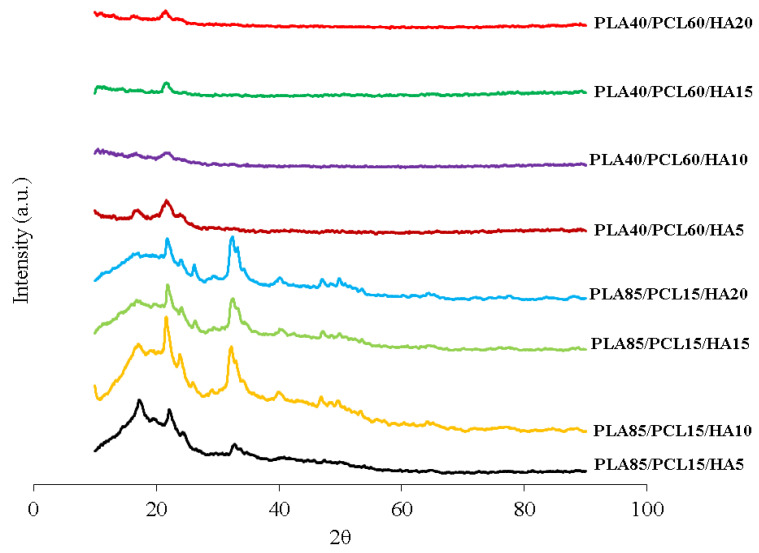
XRD patterns of the PLA/PCL/HA biocomposites.

X-ray diffraction (XRD) was utilized to identify the biocomposite’s crystallized phases (Figure 4). The 2-theta values of 31.773, 32.196, and 32.902 reflect the HA peak based on JCPDS data number 09-0432. Peaks at 2-theta values of 16.528 and 16.687 suggested the presence of PLA in biocomposites (JCPDS number 00-054-1917). The peak that indicates the existence of PCL is denoted by the 2-theta values of 21.500 and 23.900. According to the findings of this study, the XRD diffractogram showed that the PLA peak decreased with increasing HA concentration in the PLA85/PCL15 specimen. Furthermore, the PCL peak did not change significantly with increasing HA content, except for the PLA85/PCL15/H10 biocomposite, which showed the strongest PCL peak. The findings of this study show that peaks of HA can be clearly shown on biocomposite specimens made with PLA85/PCL15, and the peak intensity of HA increases with increasing HA concentration [10,14,39,43].

The findings of this research are aligned with research performed by Moura et al. [10], which compared PLA/PCL without HA and with 5% HA. The XRD diffractogram showed that the peaks of PLA and PCL decreased as the HA concentration increased, whereas the peaks of HA increased, especially at 2ϴ = 32.2. However, in specimens using 10% HA (.wt%), the PCL peak mainly increased at 2ϴ = 21.5 [10].

A different phenomenon was shown in biocomposites with PLA40/PCL60. This variation shows the low crystal phase in all variations of HA concentration, indicating that the addition of HA with a concentration of 5–20% mass inhibited crystallization in the polymer matrix. Furthermore, with the addition of HA, all composites showed wider angle diffraction peaks. This indicates that the composites with PLA40/PCL60 are amorphous. Obviously, the intensity of the diffraction peaks of PLA and PCL in the composites did not increase with increasing HA content; instead, they became smaller and wider. The reason for this may be that the addition of HA inhibits the regulation of the molecular order of PLA and PCL, thereby inhibiting crystal formation [44]. In general, PCL has a lower molecular weight than PLA. Therefore, the peak intensity of PLA decreased with increasing PCL content in composites containing PLA40/PCL60. This occurs because the increased PCL content inhibits the formation of PLA spherulites. When PCL levels increase, the molecular movements of PLA chains can be slowed down or stopped, and PLA spherulites cannot be formed [45,46,47]. The findings of this investigation are consistent with the study presented by Pires et al. [48]. In addition, the researchers stated that the addition of 30% glass mass inhibited crystallization in the polymer matrix. As a result, the crystallinity of PCL–bioglass composites is lower than that of pure PCL. This behavior is a result of filler and matrix interfacial interaction. At high concentrations, bioglass prevents polymer molecules from moving around. This makes the polymer less crystalline or amorphous [48].

**Figure 5:**
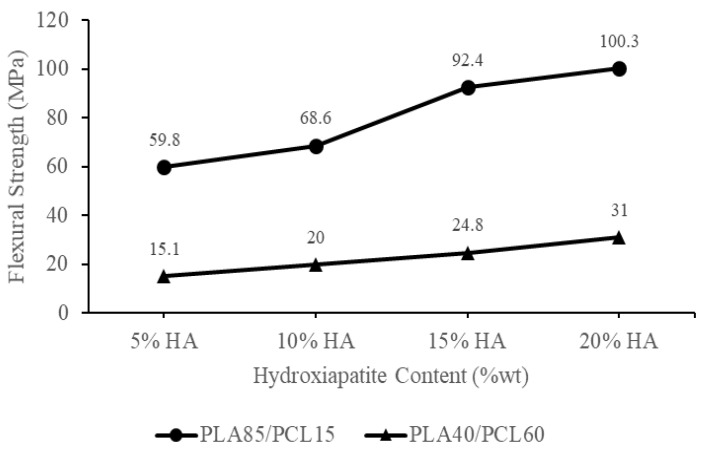
Flexural strength of the PLA/PCL/HA biocomposites.

**Figure 6:**
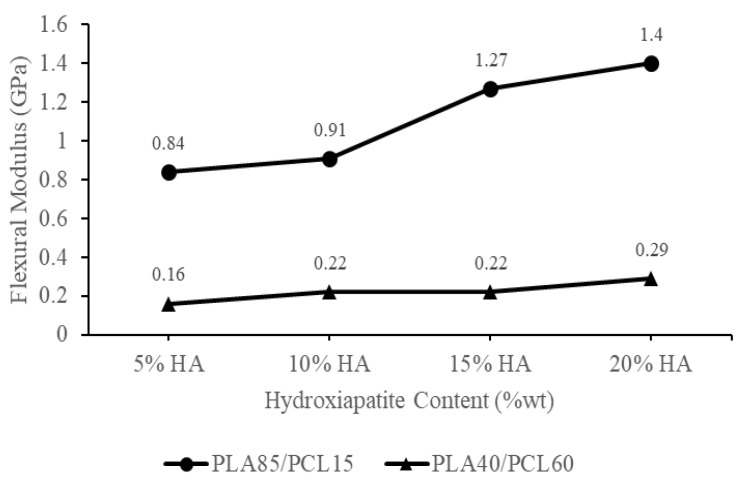
Flexural modulus of the PLA/PCL/HA biocomposites.

Figure 5 shows the flexural strength of each biocomposite specimen produced using HA, PCL, and PLA from green mussel shells. The rise in HA and PLA content raised the biocomposite samples’ flexural strength. The PLA85/PCL15/HA20 specimen had the highest flexural strength of 103 MPa. Meanwhile, the lowest flexural strength was found in the PLA40/PCL60/HA5 specimen, which was 15.1 MPa. Figure 6 shows the flexural modulus of each biocomposite specimen produced utilizing PCL, PLA, and HA from green mussel shells. The PLA40/PCL60/HA5 composition has the smallest flexural modulus of 0.16 GPa, while the composition of PLA85/PCL15/HA20 produced the highest flexural modulus of 1.4 GPa. The flexural strength and flexural modulus of the biocomposite gradually increased with increasing PLA and HA content.

The biocomposite saw an increase in flexural strength and flexural modulus, along with an increase in PLA and HA concentrations. This is because the ability to absorb energy in increasing the PCL composition decreases, and the increase in the amount of HA inhibits the ability of the polymer to flow effectively so that the polymer mixture can form a strong bond. Energy absorption is related to the mechanical properties of a material [47]. These results are consistent with a study carried out by Ferri et al. [47], in which PCL and PLA are mixed. As the PCL composition rises, the flexural modulus and flexural strength of the specimen will drop. The findings of this research concur with Charles et al. [49], who created PLA/PCL/HA biocomposites for their study. According to the findings of the flexural test, the value of the flexural modulus of the composites was found to increase with increased HA content [49].

The enhanced flexural strength of the biocomposite was also due to the establishment of an appropriate crystalline phase composition [50]. The higher the HA concentration employed in this study, the stronger the HA peak intensity on the XRD diffractogram, especially in specimens using PLA85/PCL15. The HA peak intensity is highest in the PLA85/PCL15/HA20 biocomposite. This increase in XRD peak intensity indicates that the sample has a high degree of crystallinity [51]. The biocomposite’s interfacial adhesion increased as the crystallinity increased. As a result, crack initiation in the biocomposite was prevented by increasing stress at the knee point, resulting in enhanced flexural strength [52].

The outcomes of this study are identical to those obtained by Åkerlund et al. [45]. As PCL has a toughening impact on PLA, the researchers found that increasing the PCL content decreased the compressive stress. This was because increasing the PCL content toughened the PLA. The compressive strength of biocomposites containing 15% HA decreases as PCL content increases. Other than this, the addition of HA to the PLA/PCL blend at varying percentages resulted in an increase in compressive strength, indicating that the biocomposite material became rigid [45].

PLA and HA are both brittle materials, which limits their utility in load-bearing bone approaches [45,53]. Increasing the toughness of a PLA material can be accomplished by incorporating plasticizers, stiff fillers, and copolymers into the formulation [28,42,45]. Polycaprolactone (PCL) is biocompatible and bioresorbable polyester. The incorporation of PCL in PLA has been proven to increase the resilience of brittle PLA, making it more suitable for use in bone scaffolding and bone tissue engineering applications [54]. Therefore, it is important to add HA to enhance the polymer matrix material bioactivity produced from a blend of PCL and PLA. 

The incorporation of HA into the PCL and PLA blend has the potential to improve the bioactivity and biocompatibility of the material, making it more effective at promoting bone formation and enhancing tissue adhesion [14,27,45].

As a result of the incorporation of HA particles, the mechanical properties of the PLA/PCL/HA biocomposite were improved [25]. Furthermore, the PLA/PCL/HA biocomposite is now more rigid and can withstand a greater amount of bending force. The addition of HA to the material causes it to become more rigid and resistant to plastic deformation. As a result, when a bending force is applied, the PLA/PCL/HA structure resists falling for a greater amount of time, particularly in a composition that contains a greater amount of HA. In addition, the incorporation of HA particles into the PLA/PCL blend resulted in an increase in the material’s elastic deformation range. As a result, it prevented the material from entering the zone of permanent deformation (plastic) [25]. The ability of a material to resist deformation under loads is called its flexural modulus. This is measured by how stiff the material is when a force is applied perpendicular to the long edge of a sample [23]. Heimbach et al. [22] and Ferri et al. [24] also confirmed that the flexural modulus increased (stiffness increased greatly) as the HA content increased.

The findings of this research show that the biocomposite material’s flexural strength fulfills the standards for cortical bone, which are 50–150 MPa [24]. Additionally, this research created a biocomposite material with a flexural modulus that met the requirements for the cortical bone’s flexural modulus, which is 0.6–2.69 GPa [55]. Additionally, the biocomposite created in this work has a higher flexural modulus than cancellous bone, which has a flexural modulus of 0.05–0.5 GPa [24]. The matrix’s overall modulus rises as a result of the incorporation of HA powder, since it slows down matrix phase movement around individual particles [56]. Apart from this, the flexural strength and flexural modulus produced by the biocomposite material increase with the amount of HA that is present.

According to the findings of this bending test, the flexural modulus and flexural strength of the biocomposite are influenced by the polymer composition factor and the inclusion of HA. Mechanical information on biocomposites comprising PLA/PCL/HA and relevant materials from past research are given with our findings, indicating that the findings of this investigation present excellent mechanical properties compared to the initial material. However, the flexural strength measured in this study is slightly less than that measured by Pitjamit et al. [14], especially in PLA85/PCL15/HA20 specimens, as shown in Table 3.

**Figure 7:**
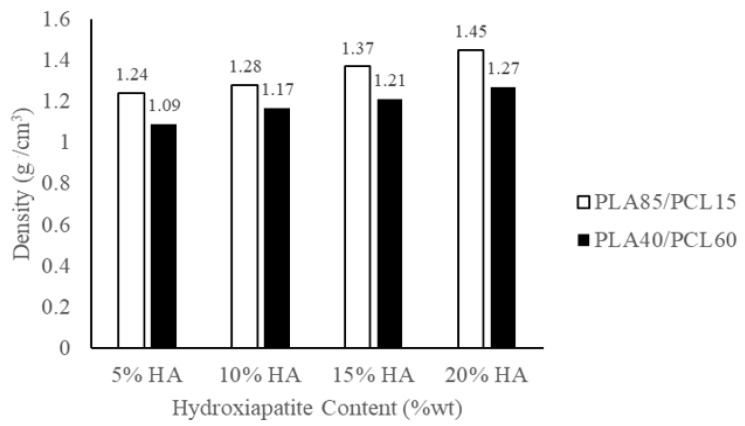
The density of the PLA/PCL/HA biocomposites.

The density of biocomposites formed from PLA, PCL, and HA obtained from green mussel shells is displayed in Figure 7. Note that Figure 7 depicts the lowest density owned by a biocomposite with a composition of PLA40/PCL60/HA5, which is 1.09 g/cm^3^, and a biocomposite owns the highest density with a composition of PLA85/PCL15/HA20 with a density value of 1.45 g/cm^3^. Biocomposite density increases as HA content rises [62]. In addition, the density of the material decreased with the decrease in the amount of PLA and the increase in the number of PCL in the biocomposite. This is due to the difference in the density values of the two polymers, where PLA has a higher density of 1.252 g/cm^3^ [63], whilst PCL has a density value of 1.135 g/cm^3^ [64]. Therefore, the addition of PLA and HA increased the density of the biocomposite specimen. As a result, the biocomposite material created in this work has a density between 1.1 and 1.3 g/cm^3^, which is comparable to the density of human cortical bone [65].

**Figure 8:**
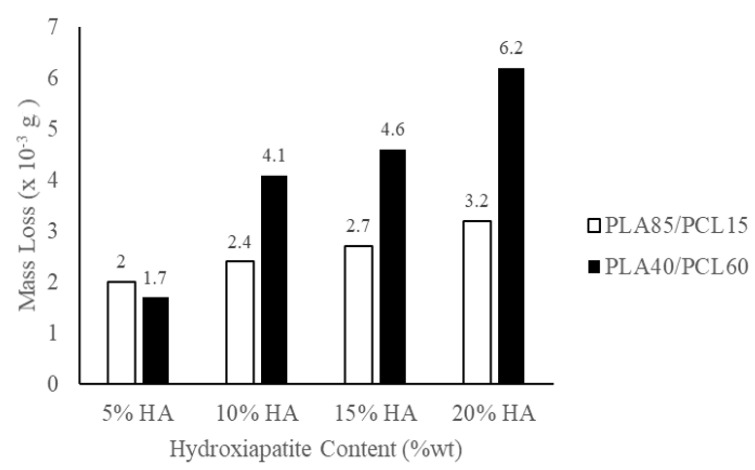
Mass loss of the PLA/PCL/HA biocomposites.

However, the biocomposites with a ratio of PLA85/PCL15/HA5 are the best candidate for medical applications compared to other variations. According to Figure 8, this occurs as a result of the biocomposites’ material degrading more quickly as more PCL and HA are utilized. Even though PLA85/PCL15/HA5 biocomposites had less robust mechanical properties than PLA85/PCL15/HA20 biocomposites, they nonetheless satisfied the standards for cortical bone. The rise in PCL composition in the biocomposites resulted in a faster rate of degradation. This is because PCL has a higher permeability than PLA. The higher the permeability, the larger the pores of the specimen so that it absorbs more water. Thus, the biocomposites which have more PCL composition will be more easily degraded [66,67].

Furthermore, with the increase in HA content, the degradation of the biocomposites accelerated. The excessive water absorption ability is caused by the increased amount of HA content used, causing an increase in hydrolysis at the fiber–matrix interface. The reduction in pH and mass loss was more distinct with greater HA content in the biocomposites, implying that increasing HA content increased the polymer surface area in contact with the immersion medium. As a result, increasing the HA content accelerates the degradation of the biocomposites.

It is known that HA is hydrophilic, and a higher HA content increases the surface wettability of the HA/polymer composite. As a result, the water infusion in the biocomposites increased, having a greater HA content, and the hydrolysis in the fiber-matrix accelerated. The structural characteristics of the biocomposites are another potential explanation for the degradation of the biocomposites, which accelerates with increasing HA content. The incorporation of HA and its agglomerates can also result in a larger absorption area for water infusion, thus, enabling faster biocomposite degradation [10,68]. The findings of this investigation are consistent with the study performed by Donate et al. [69]. Their results showed better mechanical properties resulting from a more controlled degradation rate. Furthermore, other researchers found that the rapid rate of degradation indicates more water absorption. This results in a change in the geometry of the biocomposite material. In addition, the greater the water absorption in the biocomposite material, the poorer the interface bond strength of the matrix and reinforcement, so the mechanical properties decrease [70,71,72,73,74].

The degradation behavior is a biodegradable orthopedic implant critical aspect. The preferred method of degradation is a slow, steady decline of mechanical qualities that permits the mechanical load to be gradually accepted by newly formed bones, therefore, providing the essential impetus for bone with the capacity to sustain load to be renewed. To prevent the failure of bone fixation, the fast loss of mechanical properties and implant failure (premature breakdown/deformation) must be prevented [68]. After implantation, the mechanical strength of PLA/PCL/HA decreased as a result of its rapid degradation [75]. Due to partial bone fragment immobilization, a more rapid mechanical strength decline after implantation leads to insufficient recovery [68,75,76].

**Figure 9:**
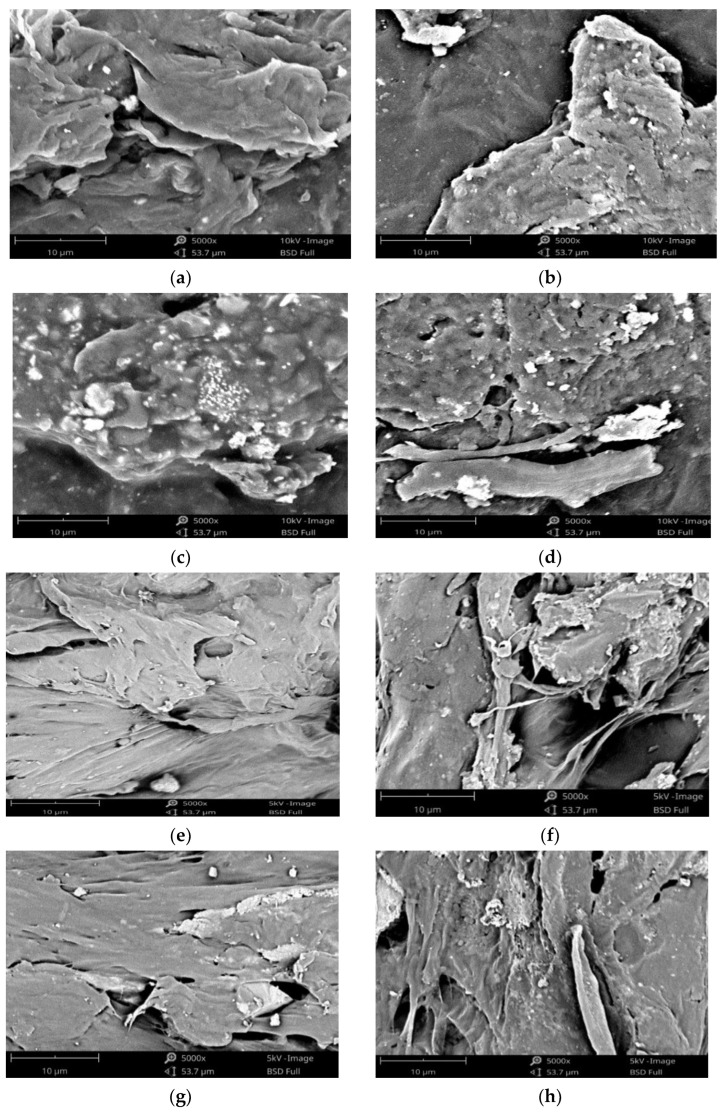
SEM images of biocomposites at 5000× magnification: (**a**) PLA40/PCL60/HA5; (**b**) PLA40/PCL60/HA10; (**c**) PLA40/PCL60/HA15; (**d**) PLA40/PCL60/HA20; (**e**) PLA85/PCL15/HA5; (**f**) PLA85/PCL15/HA10; (**g**) PLA85/PCL15/HA15; (**h**) PLA85/PCL15/HA20.

In this work, scanning electron microscope (SEM) testing has been carried out on biocomposite specimens. As illustrated in Figure 9, HA was evenly distributed throughout the biocomposite. The HA (small white particles) was well dispersed and uniform throughout the biocomposite. However, in biocomposites made with higher PCL content, the presence of HA aggregates of various sizes was seen. This occurs because the biocomposite sample with a larger PCL content increases the surface energy between PCL and HA, thereby reducing the interfacial interaction between PCL and HA and resulting in the aggregation of HA particles in the polymer matrix [14,27]. The addition of HA not only improves the bioactivity qualities of the material, but it also increases the surface roughness, which can potentially have an effect on the adhesion and proliferation of cells. Furthermore, the incorporation of HA results in the formation of a porous surface on the composite material. Cell infiltration, migration, vascularization, oxygen, and nutrient flow, as well as the omitting of waste materials, are all improved by an adequate porosity of suitable size that has interconnections between the pores [26,45]. This improves the material’s ability to endure external loading.

The ability of cells to penetrate, proliferate, and differentiate, as well as the rate at which scaffolds degrade, are all significantly impacted by the geometry and distribution of pores inside the scaffold [26].

## 4. Conclusions

The chemical blending technique has been used to produce biocomposite materials consisting of polylactic acid (PLA), polycaprolactone (PCL), as well as hydroxyapatite (HA) from green mussel shells. The occurrence of C−O, C=O, CH_2_, CH_3_, and PO_4_^3-^ peaks in the biocomposite specimens indicated the existence of PLA, PCL, and HA. The PLA and PCL peaks in X-ray diffraction (XRD) become weaker as HA concentration increases, while the HA peaks become more prominent. However, this only occurred in PLA85/PCL15 biocomposites. The XRD on the PLA40/PCL60-based biocomposite is completely amorphous. Other than that, the addition of HA inhibits the regulation of the molecular order of PLA and PCL, thereby inhibiting crystal formation in the PLA40/PCL60-based biocomposite. The use of chemical blending methods resulted in HA (small white particles) being dispersed and evenly distributed throughout the biocomposite. Moreover, when PCL was used in biocomposites in larger amounts, it led to the formation of HA aggregates of different sizes. As a consequence of having mechanical characteristics that are comparable to those of cortical bone, the PLA/PCL/HA composite material offers a great deal of promise for use in medicine, according to the findings of this study. The values of density, flexural strength, and flexural modulus increase with the amount of PLA and HA employed in biocomposite materials. In contrast, the rate of degradation increases with the amount of PCL and HA employed in biocomposite materials. The flexural modulus, flexural strength, and highest density were discovered in PLA85/PCL15/HA20 biocomposite of 1.45 g/cm^3^, 100.3 MPa, and 1.4 GPa, respectively. Meanwhile, the highest degradation rate was found in PLA40/PCL60/HA20 biocomposite with a mass loss of 6.2 × 10^−3^ g. PLA85/PCL15/HA5 biocomposite is the excellent biocomposite. The ratio of this biocomposite has complied with the requirements for human cortical bone, even though its mechanical properties are not the best. The fact that PLA85/PCL15/HA5 biocomposite degrades rather slowly in comparison to other ratios is beneficial. Due to the total immobilization of bone fragments, the mechanical strength decline after implantation happens more gradually and promotes optimum recovery.

## Figures and Tables

**Table 2:** Coding and composition of samples studied.

Polymeric Blends	Ratio (*w*/*w*)	HA (wt%)	Sample Codes
PLA/PCL	40/60	5	PLA40/PCL60/HA5
PLA/PCL	40/60	10	PLA40/PCL60/HA10
PLA/PCL	40/60	15	PLA40/PCL60/HA15
PLA/PCL	40/60	20	PLA40/PCL60/HA20
PLA/PCL	85/15	5	PLA85/PCL15/HA5
PLA/PCL	85/15	10	PLA85/PCL15/HA10
PLA/PCL	85/15	15	PLA85/PCL15/HA15
PLA/PCL	85/15	20	PLA85/PCL15/HA20

**Table 3:** Mechanical properties of the materials.

Materials	Flexural Strength (MPa)	Ref
Polylactic acid: PLA	72.3–108	[14,57,58]
Polycaprolactone: PCL	7.2–29	[14]
PLA30/70PCL	8	[42]
PLA/30HA	54	[57]
PLA/5HA	75.275	[59]
PLA/5HA	84	[60]
PCL/20HA	7.2	[61]
PLA85/15PCL/20HA	97.7	[28]
PLA85/15PCL/15HA	82.8	
PLA85/15PCL/10HA	61.3	
PLA85/15PCL/5HA	56.6	
PLA/PCL/15HA 3D-Printed	101.10	[14]
PLA/PCL/5HA 3D-Printed	101.21	
PLA40/PCL60/HA5	15.1	Current study
PLA40/PCL60/HA10	20	
PLA40/PCL60/HA15	24.8	
PLA40/PCL60/HA20	31	
PLA85/PCL15/HA5	59.8	
PLA85/PCL15/HA10	68.6	
PLA85/PCL15/HA15	92.4	
PLA85/PCL15/HA20	100.30	

## Data Availability

Data are contained within the article.
